# Preclinical Study of Locoregional Therapy of Hepatocellular Carcinoma by Bioelectric Ablation with Microsecond Pulsed Electric Fields (μsPEFs)

**DOI:** 10.1038/srep09851

**Published:** 2015-04-30

**Authors:** Xinhua Chen, Zhigang Ren, Chengxiang Li, Fei Guo, Dianbo Zhou, Jianwen Jiang, Xinmei Chen, Jihong Sun, Chenguo Yao, Shusen Zheng

**Affiliations:** 1 The Department of Hepatobiliary and Pancreatic Surgery, The First Affiliated Hospital, School of Medicine, Zhejiang University, Hangzhou, Zhejiang, 310003, China; 2 Collaborative Innovation Center for Diagnosis and Treatment of Infectious Diseases, Hangzhou, Zhejiang, 310003, China; 3 The State Key Laboratory of Power Transmission Equipment & System Security and New Technology, Chongqing University, Chongqing, 400030, China; 4 The Department of Pharmacy, Shandong University of Traditional Chinese Medicine, Jinan, Shandong, 250014, China; 5 The Department of Radiology, Sir Run Run Shaw Hospital, School of Medicine, Zhejiang University, Hangzhou, Zhejiang, 310003, China

## Abstract

Unresectable hepatocellular carcinoma (HCC) needs locoregional ablation as a curative or downstage therapy. Microsecond Pulsed Electric Fields (μsPEFs) is an option. A xenograft tumor model was set up on 48 nude mice by injecting human hepatocellular carcinoma Hep3B cells subcutaneously. The tumor-bearing mice were randomly divided into 3 groups: μsPEFs treated, sham and control group. μsPEFs group was treated by μsPEFs twice in 5 days. Tumor volume, survival, pathology, mitochondria function and cytokines were followed up. μsPEFs was also conducted on 3 swine to determine impact on organ functions. The tumors treated by μsPEFs were completely eradicated while tumors in control and sham groups grew up to 2 cm^3^ in 3 weeks. The μsPEFs-treated group indicated mitochondrial damage and tumor necrosis as shown in JC-1 test, flow cytometry, H&E staining and TEM. μsPEFs activates CD56+ and CD68+ cells and inhibits tumor proliferating cell nuclear antigen. μsPEFs inhibits HCC growth in the nude mice by causing mitochondria damage, tumor necrosis and non-specific inflammation. μsPEFs treats porcine livers without damaging vital organs. μsPEFs is a feasible minimally invasive locoregional ablation option.

Hepatocellular carcinoma (HCC) is the sixth most common malignancy worldwide^1^,^2^. It is the fifth most common malignant disease in men and the eighth most common in women. It is the third most common cause of death from cancer, just after lung and stomach cancer^1^,^2^,^3^. More than 600,000 people die of HCC each year, and at least half deaths worldwide occur in China alone^3^,^4^. HCC showed a poor prognosis because it is often diagnosed in the late stage when surgical resection is impossible^3^,^5^. Liver transplantation (LT) is currently established as the best therapy for HCC. But limited by the donor organs, many patients dropped off from the organ waiting list^6^.

The advanced HCC exceeding the UCSF/Milan criteria can be down-staged to fit the criteria using locoregional therapy such as trans-arterial chemoembolization (TACE) or percutaneous radiofrequency ablation (RFA)^6^. Beside the advantage of winning the opportunity of liver transplantation, these patients who have had successful downstage treatment also show excellent tumor-free and overall survival rates, similar to fit-criteria patients^7^. There was a significant correlation between the tumor necrosis percentage and disease-specific survival rate. Among patients whose tumors initially exceeded UCSF criteria, the extensive locoregional therapy induced tumor necrosis and thus decreased recurrence rates^8^. However, TACE has side effects from chemotherapy^9^. RFA may lead to incomplete tumor ablation in large tumors^10^,^11^ and heating sink effect of nearby vessels^10^,^11^. Therefore, the novel locoregional therapies without chemotoxicity or thermal effect are urgently needed for non-resectable HCC.

Microsecond pulsed electric fields (μsPEFs) has been used successfully in the treatment of skin tumors^12^. It is termed as bioelectric ablation, serving as another efficient downstage option for HCC. This study evaluated the anti-neoplastic effects and investigated its possible molecular mechanism.

## Methods

### Animals and cell line

Human hepatoma cell line (Hep3B) was purchased from Shanghai Institute of Cell Biology, Chinese Academy of Sciences (Shanghai, China). Male nude mice (weight 18–20 g, 4–5 weeks old) were purchased from Shanghai Laboratory Animal Center (Shanghai, China). The mean weight of three female domestic swine was 65 kg. Animals were housed in a Special Pathogen Free Laboratory Animal Center. Animal protocols were approved by Experimental Animal Care and Ethics Committee of Zhejiang University and Chongqing University. The methods were carried out in accordance with the approved animal experiment protocol of Zhejiang University and Chongqing University.

### Pulse generator, electrode and the pulsed electric field distribution

**Figure 1:**
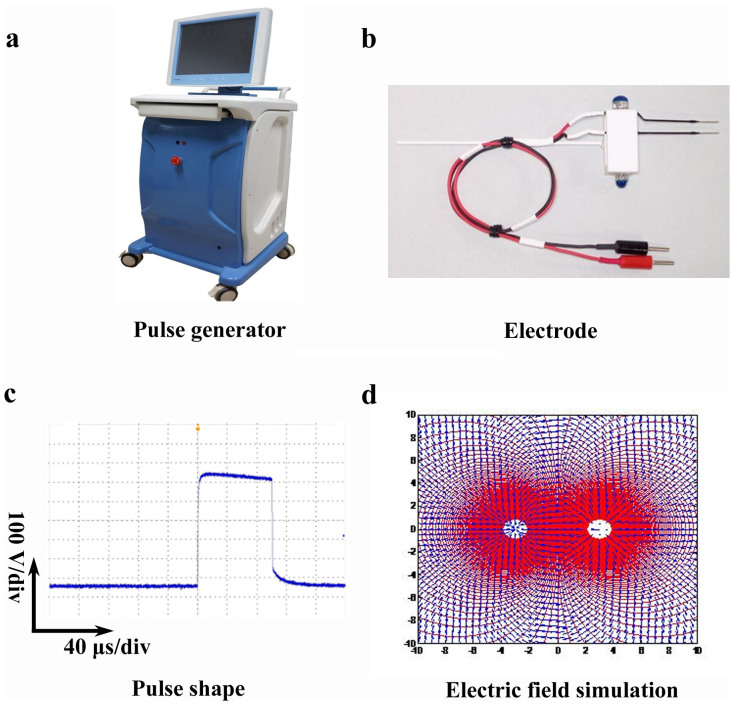
Pulse generator, electrode, electric field simulation and typical waveform of pulses during treatment. (a) Microsecond pulse generator was developed by State Key Laboratory of Power Transmission Equipment and System Security and New Technology, Chongqing University. Amplitude of output pulse voltage could reach up to 3 kV with the rise time at ns level, pulse width at μs level and the repetition rate of 1 ~ 100 Hz. The voltage amplitude and pulse width as well as repetition frequency is adjustable. (b) Two-needle electrode (10 mm-Tip, 0.5 mm diameter, variable gap from 1 mm to 20 mm) was bought from NEPA GENE Company, Japan. (c) Typical waveform of pulses during the application of μsPEFs. Pulses delivered to the tumors with the application of two-needle electrode, and DPO4054 real-time oscilloscope (Tektronix Company, U.S.) was used to monitor the waveform of pulses. (d) The electrode placement and electric field exposure simulation. The two white dots in the middle are the tips of needle electrode. The red area is full coverage overlapped by the electric field delivered by two electrodes. The scale showed the distance in mm.

Microsecond pulse generator was designed and made by State Key Laboratory of Power Transmission Equipment and System Security and New Technology, Chongqing University (Figure 1a). Two-needle electrode (10 mm-Tip, 0.5 mm diameter, variable gap from 1 mm to 20 mm) was bought from NEPA GENE Company, Japan (Figure 1b). Typical waveform of pulses during the application was shown in Figure 1c. Pulses delivered to the tumors with the application of two-needle electrode, and DPO4054 real-time oscilloscope (Tektronix Company, U.S.) was used to monitor the waveform of pulses (Figure 1c). Microsecond pulses (2.5 kV, 100 μs, 1 Hz, 90 pulses) were applied to tumor bearing mice twice in 5 days with 4-day intervals.

### Electrode placement

Two needle electrodes were placed parallelly to each other. Image reconstructions along the electrode axis were performed to measure distance between the tips of the electrodes. The needle electrode placement is evaluated by a self-designed electric field exposure simulation model that can predict the ablation area as shown in Figure 1d.

### Parameter setting

The voltage applied was 3000 V and a current 50 amperes. The totals of 90 pulses were delivered in 1 Hz to the tumor in mice. In swine, the same treatment was repeated after 4 days (two treatments in 5 days with 4 day-intervals). The energy was transmitted via the bipolar electrodes into the target tumor in the form of 100 microsecond electrical impulses. All the interventions were performed under ultrasound guidance and animal anaesthesia.

### Tumor ablation effect observation

10^6^ human HCC Hep3B cells were injected subcutaneously on nude mice to set up HCC tumor-bearing animal model. 48 mice were randomly divided into three groups. Control group (n = 16): nude mice had no μsPEF treatment except anesthesia; Sham group (n = 16): nude mice were given anesthesia and the tumors were punctured with needle electrode but no μsPEF treatment; μsPEFs treated group (n = 16): nude mice were given anesthesia, punctured with electrode and treated with μsPEFs. The mice in each group were randomly divided into subgroup A (n = 8) for survival follow-up and subgroup B (n = 8) for sample collecting. Throughout the treatment, the mice were anesthetized and positioned on a warming stage. The animal weights and tumor growth were followed up.

### Animal safety evaluation

The safety evaluation of μsPEFs treatment *in-vivo* was conducted on 3 swine with percutaneous approach by ultrasound guide. μsPEFs were delivered to porcine liver tissue (2.5 kV, 100 μs, 1 Hz, 90 pulses) to observe the impact on the vital organs. The animals were monitored for temperature, pulse, respiration, heart beat and ECG. Animals were monitored for 24 hours and then euthanized. All procedures were performed under isoflurane inhalation anesthesia.

### Sample collections

On the 3^rd^ day post treatment, the mice in subgroup B were euthanized and sampled for H&E staining, transmission electron microscopy (TEM), immunohistochemistry (IHC) and flow cytometry. The blood sample from portal vein was collected to measure plasma cytokines.

### Tumor volume and animal survival follow-up

All mice in the subgroup A were followed up for survival study marked by the survival elongation ratio (SER). SER was calculated according to the reference^13^ described: SER = (T1−T2)/T2 *100%, where T1 and T2 are survival time of the treated group and the control group, respectively. The tumors of mice in the subgroup A were measured daily. Tumor volume was calculated as the reference^14^ described: V = 0.52 × D1^2^ × D2, where D1 and D2 are short and long tumor diameters, respectively.

### Tumor necrosis identification by histopathology and TEM

The dissected tumor was fixed in 40 g/L neutral formaldehyde and embedded in paraffin, cut into 3 μm slices, stained with hematoxylin and eosin (H&E), and then observed under light microscopy. Meanwhile, the tumor tissue was processed by standard procedures for TEM as previously described^15^,^16^. Briefly, tumor tissue was fixed in 2.5% glutaraldehyde (4°C, pH 7.4), postfixed in 1% osmium tetroxide, and embedded in an epon–araldite mixture. Ultra-thin sections of the tissues were prepared and placed on mesh copper grids and stained with uranyl acetate and lead citrate. The ultrastructure of tumor was analyzed on a Philips Tecnai 10 electron microscope (Philips, Eindhoven, Netherlands).

### Immunohistochemical staining

Immunohistochemistry localized the expression of proliferating cell nuclear antigen (PCNA), CD56 and CD68 of a total of 24 tumor specimens (8 controls, 8 sham and 8 μsPEFs treated). IHC was performed with rabbit monoclonal antibodies to PCNA (AbCam, Cambridge, UK), CD56 (AbCam, Cambridge, UK) and CD68 (AbCam, Cambridge, UK) according to our previous protocol^17^. IHC results were recorded by light microscopy and analyzed by Image-Pro Plus (Media Cybernetics, Crofton, MA, USA). The cell nuclei or membrane was stained yellow or brown suggesting the positive signal. The protein expressions were quantified by integrated optical density (IOD) per high-powered field (hpf). Data were presented as the average result in ten randomly selected fields.

### Tumor cell isolation

The tumor tissues were collected and digested with collagenase (Sigma, 40 U/ml) in RPMI 1640 for 60 min at 37°C, and then cell suspension was filtered. Cell suspension was centrifuged at 800 × g for 5 min and the pellets were rinsed by RPMI 1640. The tumor cells were suspended in RPMI 1640 and then 0.5 ml cell suspension was counted with a cell viability analyzer (Vi-cell, Backman).

### Quantitative detection of cell necrosis and apoptosis using flow cytometry

Annexin V-FITC Apoptosis Detection Kit (BD Biosciences) was used to detect cell apoptosis or necrosis as our previous protocol^17^. Annexin-V and Propidium Iodide (PI) double staining was used to evaluate viable cells (Annexin-V−/PI−), apoptotic cells (AnnexinV+/PI−) and necrosis cells (AnnexinV+/PI+). 1 × 10^5^ tumor cells were suspended in 100 μL HBSS containing fluorescein isothiocyanate (FITC) staining Annexi V (RD, USA) and phycoerythrin (PE) conjugated propidium iodide (PI) (RD, USA) to identify apoptosis and necrosis, respectively. Fluorescent dyes Annexin V and PI were diluted to 1 μg/ml in HBSS containing 1% FBS, incubated 30 min on ice. After staining, the cells were rinsed twice in HBSS/1% FBS and then run in a flow cytometry (LSR, BD). The data were analyzed by Flow Jo 7.6.1 Software (Flow Jo Systems, Treestar, USA). The percentages of cell apoptosis or necrosis were calculated.

### Detection of mitochondrial membrane potential (Δψ)

Mitochondrial trans-membrane potential was detected with flow cytometer. Briefly, the isolated tumor cells were incubated in 24-well plates and then pelleted, washed with PBS and suspended in 0.3 ml diluted mitosensor reagent (1 μmol/ml in incubation buffer) for 20 min, then 0.2 ml incubation buffer was added. Cells were centrifuged and then suspended in 40 μl incubation buffer. Finally, the cells were washed and suspended in 1 ml PBS for flow cytometry analysis (LSR, BD) for JC-1(5, 5′, 6, 6′–tetrachloro-1, 1′, 3, 3′-tetraethylbenzimidazolylcarbocyanine iodide) changes. JC-1 is a dye for measuring membrane potential of mitochondria. The mitosensor dye JC-1 aggregates in the mitochondria of normal cells and emits red fluorescence against a green monomeric cytoplasmic background staining. In the damaged cells with the collapsed mitochondria, JC-1 is not able to accumulate in the mitochondria and remains as monomers emitting green fluorescence.

### Plasma cytokines measurement

Tumor necrosis factor (TNF-α), IL-6, IL-10, tumor growth factor (TGF-β1) and vascular endothelial growth factor (VEGF) were measured with the enzyme-linked immunosorbent assay (ELISA) (RD, USA) according to manufacturers’ protocols. The results were expressed as nanogram per liter serum (ng/L).

### Apoptosis test by cleaved Caspase-3

The key executioner on apoptosis pathway, cleaved Caspase-3 was tested by immunofluorescence staining. The tumor sections were de-paraffinized and then immerged in citrate buffer for 5 minutes to retrieve antigen, 3% hydrogen peroxide 10 minutes to inactivate endogenous peroxidase, blocked with goat serum, incubated in a humidity tray with antibodies against cleaved caspase-3 at Asp175 (Beyotime, Shanghai, China, 1:100) for 2 h at room temperature. After washing 5 times in PBST tumor secions were incubated in a secondary antibody, Alexa Fluor-488-labeled goat anti-rabbit IgG (Invitrogen, 1:250) for 30 min at room temperature in darkness. Cover slips were mounted with mounting media (Vector Laboratories, H-1200), which contained DAPI to identify the nuclei. The number of positive cells was scored by counting of three sets of at least 100 cells under the microscope. Each experiment was performed twice.

### Statistical analysis

Data were presented as mean ± standard error (SEM). Statistical analyses were performed with the software package SPSS for Windows (version 17.0; SPSS, Inc., Chicago, IL, USA). The survival distribution function was evaluated by the Kaplan-Meier survival curve and Students T-Test. Cell apoptosis/necrosis and JC-1 results were analyzed by Flow Jo 7.6.1 Software. IHC results were analyzed by Image Pro-Plus software. A p-value of less than 0.05 was considered statistically significant.

## Results

### *In vivo* efficacy of μsPEFs against Hep3B xenografts

**Figure 2:**
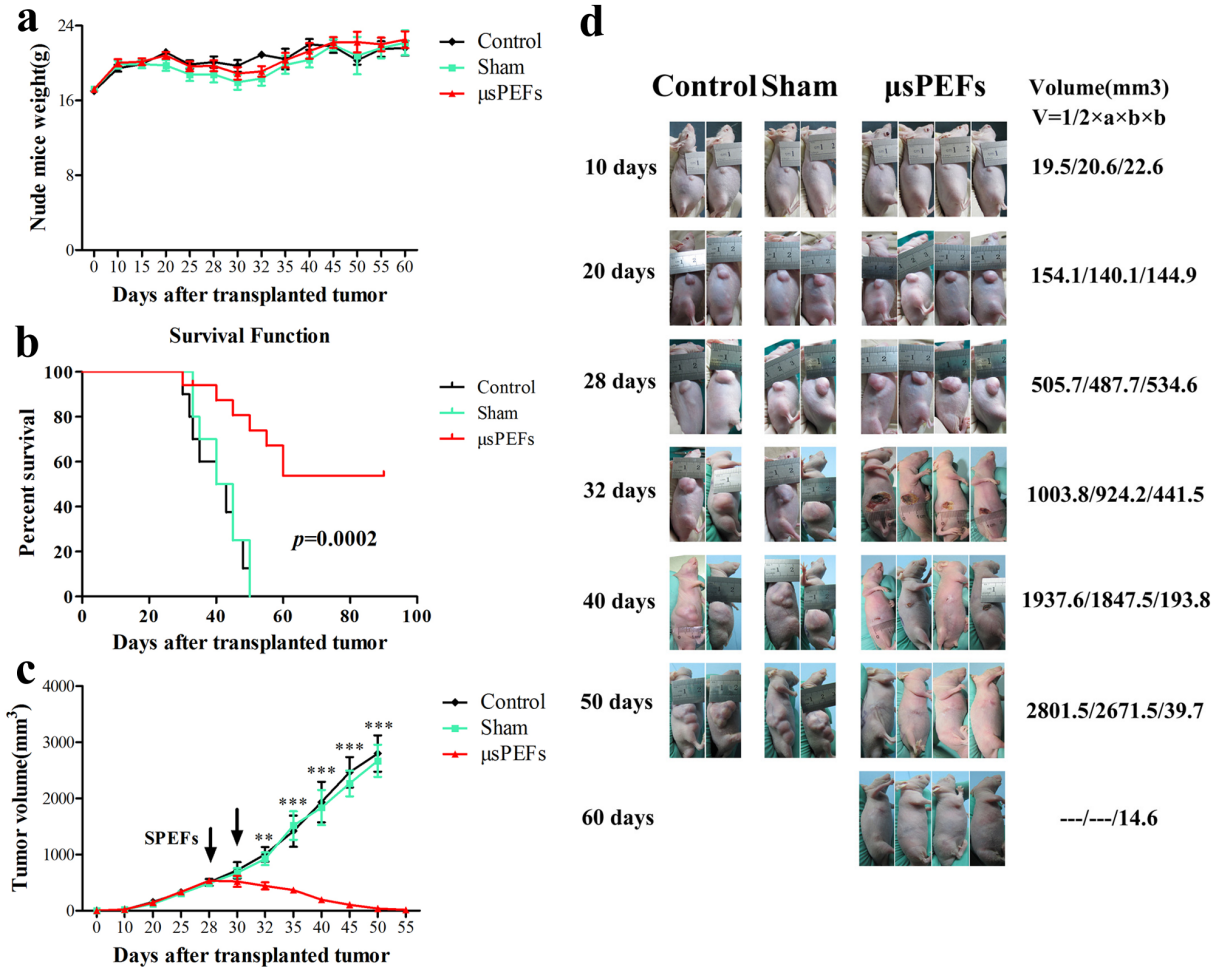
Robust efficacy of μsPEFs against Hep3B xenografts in nude mice. (a) The weights of nude mice from the different groups at different time points after transplanted tumor. The weight was present as mean ± SEM, n = 8. (b) Kaplan-Meier survival curve of nude mice implanted with Hep 3B tumor in the different groups (n = 8). (c) The changes of tumor volume in nude mice from the different groups at different time points after transplanted tumor. The tumor volume was presented as mean ± SEM, n = 8. **p < 0.01 or ***p < 0.001 indicated significant differences in μsPEFs treated group versus the control and sham groups. The arrows indicated twice μsPEFs on day 28 and day 30. (d) The dynamic process of μsPEFs ablation tumor compared with the control and sham groups. The volume presented as mean tumor volume (mm^3^) at each time point.

There was no significant weight loss in μsPEFs treated group compared with sham or control group (Figure 2a). The Kaplan-Meier survival curve showed that the μsPEFs treated group gained the significantly longer survival versus the sham and control groups (p = 0.0002) (Figure 2b). The tumor volume was measured before and after ablation. Tumor volume in the μsPEFs group decreased after the μsPEF treatments compared with the sham and control (Figure 2c), suggesting that μsPEFs significantly inhibited tumor xenografts in nude mice.

### μsPEFs treatment induced substantial necrosis of tumor cells in Hep3B xenografts

**Figure 3:**
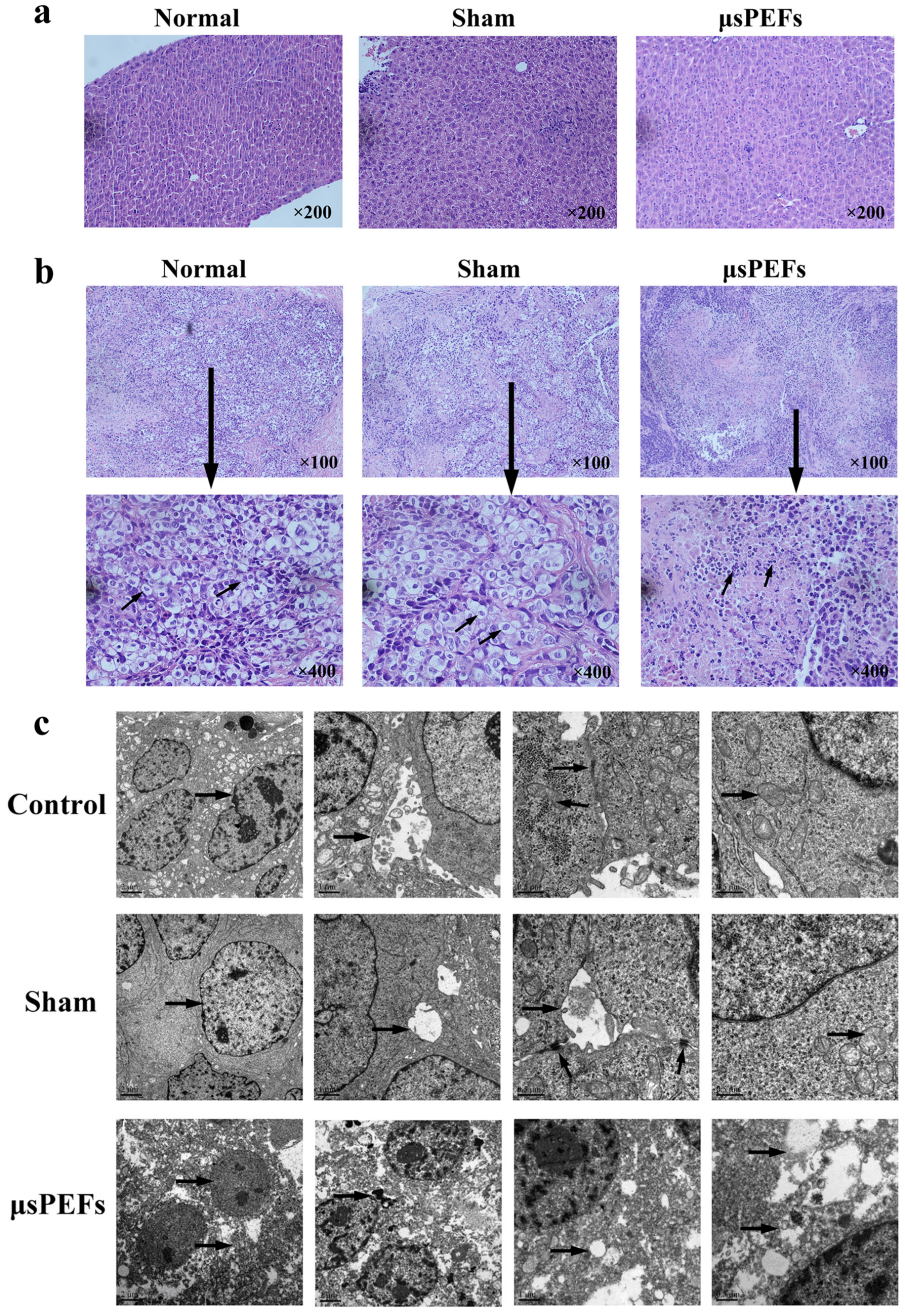
μsPEFs treatment induced substantial necrosis of tumor cells in Hep3B xenografts. (a) Liver histopathology structure of nude mice in the different groups. Magnification: 200×. (b) The representative tumor histopathology in Hep3B xenografts. The arrows indicated variations of tumor cells in the different groups. Magnification: 100× & 400×. (c) The representative tumor ultra-structure in Hep3B xenografts by TEM. The arrows indicated variations of nuclear, mitochondria, other organelles and tight junction in tumor cells from the different groups.

To analyze the morphologic characteristics of tumor cells after μsPEFs treatment, tumor histopathology and ultra-structure in different groups were compared. First, we compared liver structure of nude mice among the different groups, and found no variations in the liver presenting a normal structure with well-arranged hepatocyte cords (Figure 3a). The tumor cells in control and sham group showed prominent angular shape with intact and large vesicular nucleus and big nucleolus. In the μsPEFs treated group, the necrosis located in the electrode-covered area. μsPEFs treatment induced substantial coagulative necrosis, destruction of tumor cells with adjacent inflammatory and fibroblastic cell infiltration (Figure 3b).

Meanwhile, the ultrafine structure of tumor by TEM in the control and sham groups showed distinct cell membranes, abundant granular eosinophilic cytoplasm, round nuclei with coarse chromatin and complete nuclear membrane and prominent nucleoli. The bile capillary between tumor cells and tight junction were also noted. μsPEFs treated hepatic tumor displayed necrotic characteristics of tumor cells such as caryolysis, broken cytoplasm, damaged chromatin, poorly defined nucleus, clear vesicles in the cytoplasm and disrupted cell membrane with leaking cell contents (Figure 3c).

### μsPEFs treatment induced mitochondrial dysfunction and cell necrosis

**Figure 4:**
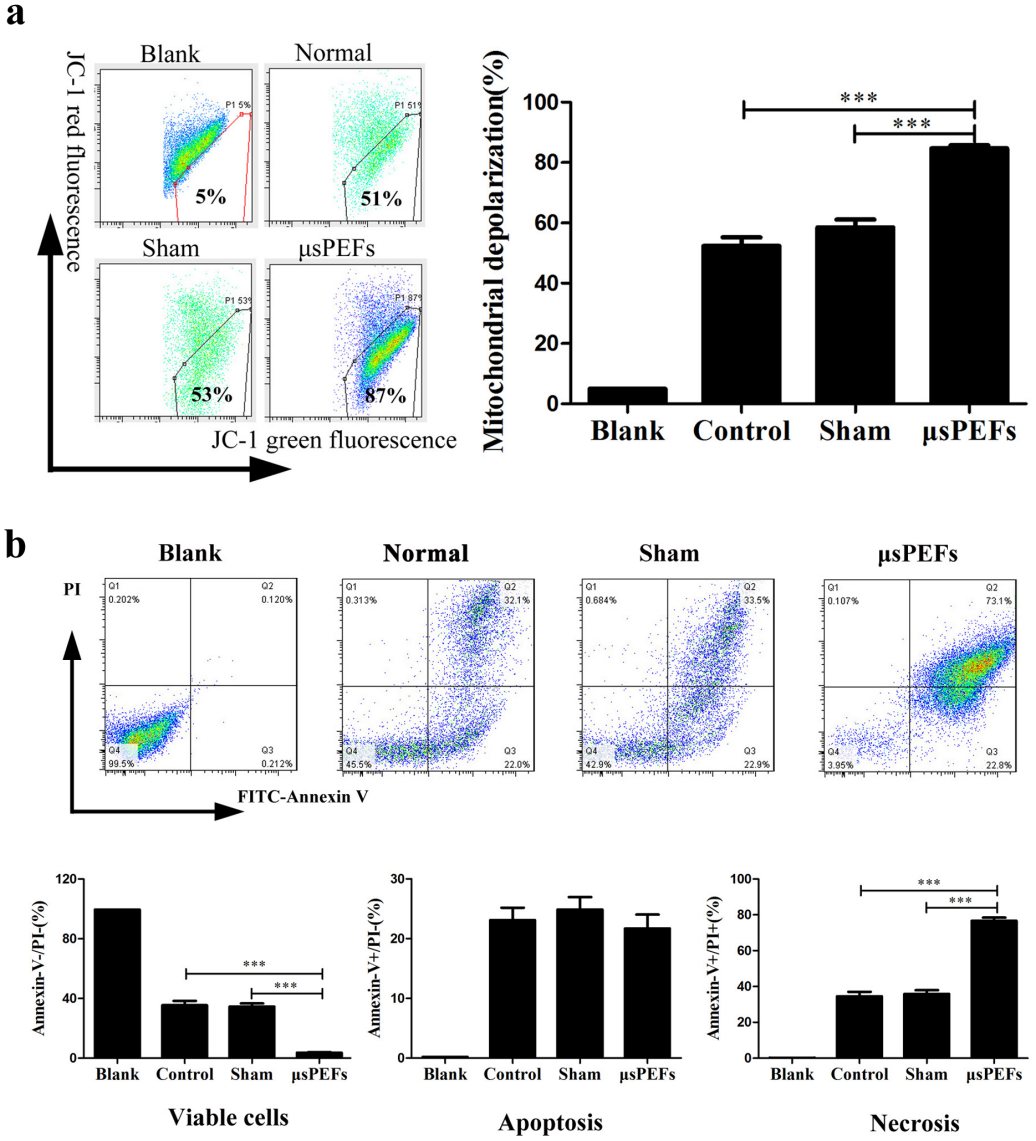
μsPEFs induced mitochondrial dysfunction and cell necrosis using flow cytometry. (a) μsPEFs induced mitochondrial dysfunction and damage by mitochondrial potential sensor JC-1. In the mitochondria of normal cells, JC-1 aggregates and emits red fluorescence, but in the damaged cells with the collapsed mitochondria, JC-1 is not able to accumulate in the mitochondria and emits green fluorescence. (b) μsPEFs induced tumor cells necrosis by flow cytometry. Annexin-V and Propidium Iodide (PI) double staining was used to evaluate viable cells (Annexin-V−PI−), apoptotic cells (AnnexinV+PI−) and necrosis cells (AnnexinV+PI+). The data were expressed as the mean ± SEM of three independent experiments.

To better understand the molecular mechanism of μsPEF ablation, mitochondrial function was detected by the mitochondrial potential sensor JC-1. In the mitochondria of normal cells, JC-1 aggregates and emits red fluorescence, but in the damaged cells with the collapsed mitochondria, JC-1 is not able to accumulate in the mitochondria and emits green fluorescence. In the detection, the percentages of JC-1 green fluorescence in the isolated tumor cells were (51 ± 5.3) % and (53 ± 6.5) % in the control and sham groups respectively, while the percentage was significantly increased to (87 ± 2.6) % in the μsPEFs treatment group (Figure 4a). These results suggest an obvious collapse in the mitochondrial Δψ, which indicated mitochondrial dysfunction and damage after μsPEFs treatment.

Meanwhile, the apoptosis and necrosis percentages of the isolated tumor cells from the different groups were tested by flow cytometry. The viable cells (Annexin V−/PI− cells) were significantly decreased in the μsPEFs group versus sham and control group (both p < 0.001). The percentages of cells apoptosis (Annexin V+/PI− cells) had no difference, while the percentage of cells necrosis (Annexin V+/PI+ cells) remarkably increased in the μsPEFs group compared with the sham and control groups (both p < 0.001) (Figure 4b).

These results demonstrated that the μsPEFs treatment induced mitochondrial dysfunction and damage of tumor cells, thereby led to tumor cell necrosis during the process of tumor ablation *in vivo*.

### μsPEFs treatment induced no obvious apoptosis

**Figure 5:**
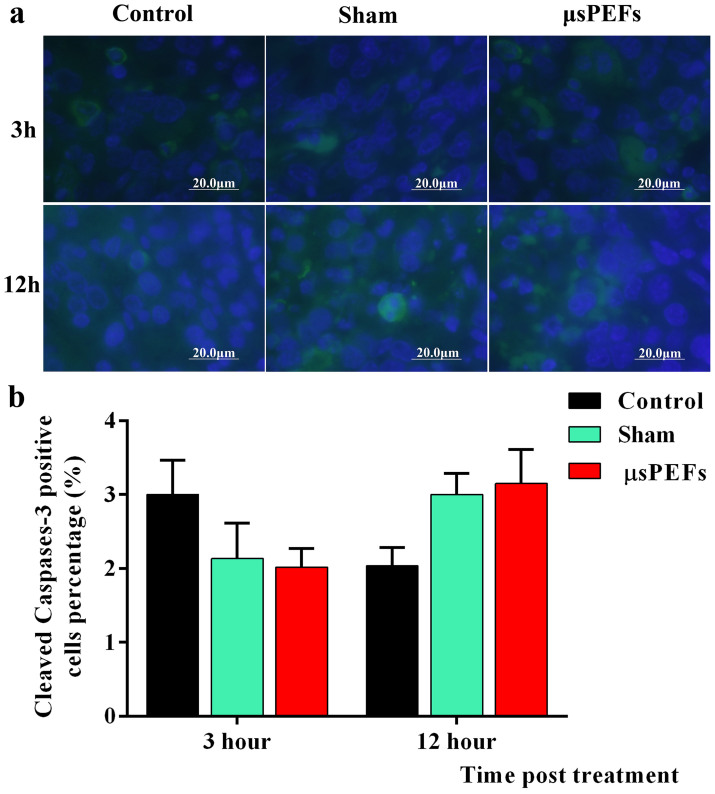
The immunohistochemical labeling of cleaved Caspase-3. (a) The cleaved Caspase-3 was labeled by Alexa Fluor-488-labeled antibody (green color) and the nuclei were stained by DAPI (blue color). (b) The number of positive cells was scored by counting of three sets of at least 100 cells under the microscope, and the percentages of Caspase-3 positive cells were used for statistical comparison. Each experiment was performed twice.

To observe whether μsPEFs treatment induce tumor cell apoptosis, the cleaved Caspase-3 was detected by immunofluorescence staining (Figure 5a). The statistical analysis showed that the percentage of cleaved Caspase-3 activation had no significant changes post electric field treatment versus control and sham tumors no matter at 3 hour or 12 hour post treatment, as shown in Figure 5b. These results suggest no existence of apoptosis characterized by cleaved Caspase-3 activation. Caspases family is inactive pro-enzymes that are only activated by proteolysis cleavage. Caspase 8 and 9 activate Caspase-3 and then Caspase-3 cleaves vital cellular proteins to initialize apoptosis. In this study, there is no proof that μsPEFs trigger obvious apoptosis.

### μsPEFs promoted inflammation and inhibited proliferation during tumor ablation

**Figure 6:**
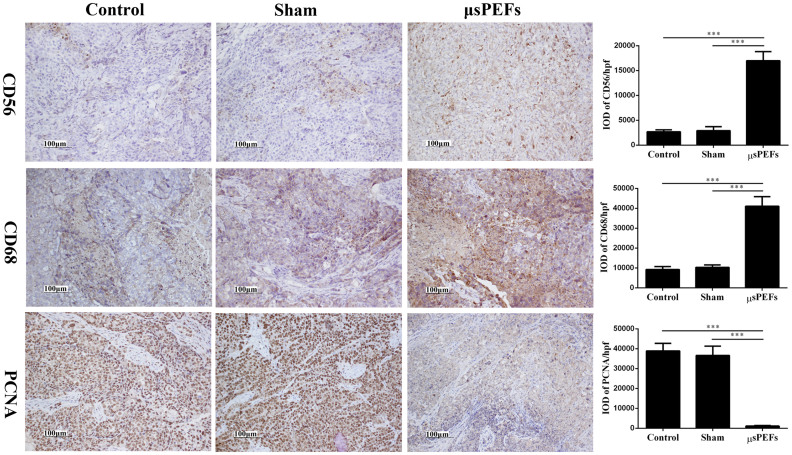
CD56, CD68 and PCNA expressions in tumor tissues after μsPEFs by IHC. The expressions of CD56, CD68 and PCNA in tumor tissues after μsPEFs by IHC were shown on the left. Quantification of IHC staining-positive areas in tumor tissues were shown on the right. The data were analyzed by Image-Pro Plus software. The cell nuclei or membrane was stained yellow or brown suggesting the positive signal. The protein expressions were quantified by integrated optical density (IOD) per high-powered field (hpf). Data were presented as the average result of ten random high-power fields. ***p < 0.001.

To observe the changes in tumor microenvironment after the μsPEFs treatment, inflammatory cells (CD56+ or CD68+) infiltration and tumor cell proliferation (PCNA+) in the treated tumor tissue were identified by IHC (Figure 6).

Protein CD56 molecular expresses on the surface of the active cells including NK cells and Schwann cells, and protein CD68 is the unique marker of macrophages. The results showed that the positive CD56 and CD68 were significantly increased in the μsPEFs group versus the sham and control groups (both p < 0.001) (Figure 6), which suggested that the μsPEFs treatment promoted inflammatory cells infiltration after tumor ablation *in vivo*.

PCNA is closely associated with DNA synthesis and plays an important role in the initiation of cell proliferation. The IHC results indicated that the expression of PCNA was significantly decreased in the μsPEFs treated group compared to the sham and control groups (both p < 0.001) (Figure 6), suggesting that the μsPEFs treatment inhibit tumor cells proliferation after tumor ablation *in vivo*.

### μsPEFs treatment increased inflammatory cytokines and decreased growth factors during tumor ablation

**Figure 7:**
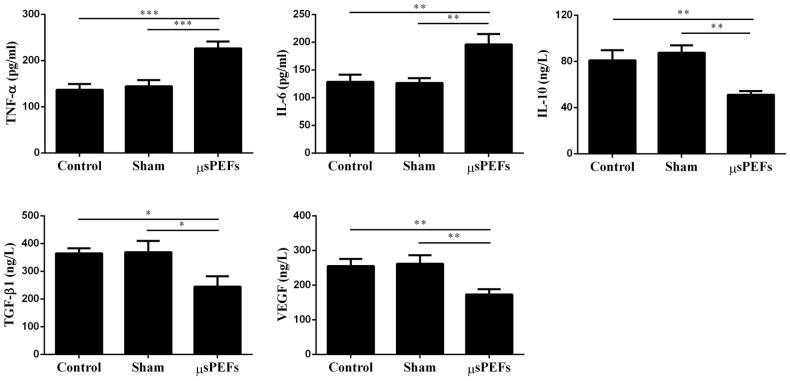
Plasma levels of inflammatory cytokines (TNF-α, IL-6 and IL-10) and growth factors (TGF-β1 and VEGF) after μsPEFs ablation tumor *in vivo*. The results were expressed as picogram per milliliter (pg/ml) or nanogram per liter serum (ng/L). The data were expressed as the mean ± SEM, n = 8. Each sample was detected for three times.

Inflammation responses play a crucial role in the process of cells necrosis and clearance, tissue degradation and tissue formation^18^. The inflammatory mediators in plasma were detected (TNF-α, IL-6 and IL-10) after the μsPEFs treatment. TNF-α and IL-6 were significantly higher in the μsPEFs treated group (226 ± 15.4 pg/ml and 196 ± 18.9 pg/ml) than those in the sham group (144 ± 13.5 pg/ml and 127 ± 8.7 pg/ml) and control group (137 ± 12.8 pg/ml and 128 ± 12.9 pg/ml) (both *p* < 0.001 and *p* < 0.01), respectively (Figure 7). In contrast, plasma level of IL-10 was obviously decreased in the μsPEFs treatment group (51 ± 3.3 ng/L) versus the sham group (87 ± 6.4 ng/L) and control group (81 ± 8.8 ng/L) (both *p* < 0.01) (Figure 7). These data indicated that μsPEFs treatment induced an increased pro-inflammatory cytokines (TNF-α and IL-6) and a decreased anti-inflammatory cytokine (IL-10) during tumor ablation *in vivo*.

Plasma tumor growth factor (TGF-α and TGF-β) and VEGF are necessary for tumor cells proliferation, vascular formation and tissue repair^18^. The results showed that TGF-β1 and VEGF in plasma were remarkably decreased in the μsPEFs group (245 ± 37.4 ng/L and 173 ± 15.2 ng/L) versus the sham group (369 ± 40.8 ng/L and 262 ± 24.9 ng/L) and control group (364 ± 18.7 ng/L and 255 ± 20.7 ng/L) (both *p* < 0.05 and *p* < 0.01), respectively (Figure 7). These results suggested that μsPEFs decreased growth factors such as TGF-β1 and VEGF during tumor ablation *in vivo*.

### μsPEFs showed no damage on hemodynamic, pulmonary and renal function, but an transient impact on liver function

Porcine vital signs were sampled and tested before treatment, during treatment, 1 hour and 24 hours after treatment. Hemodynamic and pulmonary function were monitored by ECG, pulse oximetry, mean arterial pressure, central venous pressure, core temperature, arterial carbon dioxide partial pressure, arterial oxygen partial pressure (Table 1). Renal function was monitored by urine output, blood urea nitrogen, creatinine (Table 2). There were no significant changes before and after μsPEFs treatment (all *p* > 0.05). Liver function was affected 24 hours post μsPEFs treatment indicated by significantly increased serum aspartate aminotransferase, alanine amiontransferase and alkaline phosphatase (all *p* < 0.01) (Table 2).

## Discussion

With the new ablation modalities emerging, the locoregional therapies have had a major breakthrough in the treatment of unresectable HCC. Together with radical resection, the locoregional ablation represented by RFA was adopted as the first-line therapies for HCC by the guidelines of the European Association for the Study of the Liver (EASL-EORTC)^19^. The American Association for the Study of Liver Diseases (AASLD)^20^ and the Asian Pacific Association for the Study of the Liver (APASL)^21^. In these guidelines, locoregional ablation is recommended as a strategy alternative to surgery resection. But a safety margin is emphasized that normal hepatic tissue of at least 5 mm to be resected or ablated to ensure a complete removal of HCC^22^. Incomplete ablation of RFA may change the tumor microenvironment by heating the tumor to enhance the residual tumor to grow faster and accelerate HCC metastases^23^,^24^,^25^. So the novel non-thermal locoregional ablation method is in great need. Bioelectric tumor ablation with pulsed electric fields may potentially address that limitation.

Bioelectric tumor therapies produce electrical stimuli on tumor cells. The commonly used method is the pulsed electric fields. The pulsed electric filed in microsecond can perforate the cell membrane reversibly and enhance the delivery of chemotherapeutic drugs (electroporation). The electrochemotherapy in human tumors has been approved by European Standard Operating Procedures of Electrochemotherapy (ESOPE) mainly in skin tumors^26^,^27^,^28^,^29^. When increasing the energy, the effect of microsecond pulsed electric fields on cell membrane become irreversible and can induce the cell death without combination of chemotherapy drugs^30^. Different from RFA which heats the tumor to 60–100°C, μsPEFs deliver the energy in a relatively short time period (microsecond) and therefore has not as much heat accumulated as RFA does. The HCC morphological changes after μsPEF treatment were analyzed by the light microscopy, TEM, and flow cytometry. Results demonstrated the tumors underwent mainly necrosis when they were exposed to μsPEFs without combination of any other drugs. The necrosis caused local inflammatory and fibroblastic reaction which contributes to the final clearance of the necrotic lesion.

The electrode design is the key of μsPEF application. In our experiment, a bipolar electrode is used to deliver the electric field to cover the tumor completely. It was precisely inserted the tumor and a safe margin of 5 mm of adjacent liver tissue is included. This safe margin in μsPEF treatment has been proved by the electric field simulation and pathology. μsPEFs caused non-selectively damages to normal cells and cancer cells only if they are in the targeted area covered by the electrode. The well-designed electrode can maximize the ablation of the tumor while minimizing injury to normal liver tissue.

Beside confirmation of the anti-tumor effect of μsPEF, the current study also enhances the understanding of *in vivo* mechanism of μsPEF. VEGF is a protein produced by cells that stimulates vasculogenesis and angiogenesis which HCC rely on for growth^31^. Anti-VEGF therapy with sorafenib has been approved successful as the first systemic therapy and demonstrate improved survival in patients with advanced-stage HCC^32^. H&E staining showed rich neovasculature in the control and sham tumors and IHC showed overexpression of VEGF. After μsPEF treatment, the tumors were deprived of the blood vessels and the expression of VEGF was decreased.

Moreover, CD56 and CD68 positive cells play role in local immune response in patients with HCC^33^, so the expressions of CD56 and CD68 was investigated by tumor tissue IHC. CD68, cluster of differentiation 68, is a glycoprotein which binds to low density lipoprotein. It is expressed on macrophages in liver. CD56 is prototypic marker of natural killer (NK) cell. Both NK and macrophage cells are abundant in liver lymphocytes and play an important role in first-line, innate defense against viral infection and tumor transformation^34^. The increased expressions of CD56 and CD68 indicated the activated local immune microenvironment against tumor. PCNA is a protein that acts as a progressivity factor for DNA polymerase. It expressed in the nucleus during DNA synthesis. The decreased PCNA indicated the tumor DNA synthesis was inhibited by μsPEF.

In addition, TGF is composed of two polypeptide growth factors, TGF-α and TGF-β. These proteins induce oncogenic transformation which induces the cells to proliferate and overgrow^35^. Thus, it is a promising target for anti-HCC treatment. Recent studies have shown that inhibition of TGF-β signaling results in multiple synergistic down-stream effects which will improve the clinical outcome in HCC^35^. Our results showed that μsPEF corrected the deregulated tumor suppressor genes and oncogenes. The over-expression of TGF-α and the inhibition of TGF-β was inverted by μsPEF. IL-6 is an interleukin that acts as both a pro-inflammatory cytokine and an anti-inflammatory cytokine. In current study, μsPEF damaged the tumor and caused necrosis. The local infection and hepatic trauma caused IL-6 secretion by macrophages to stimulate immune response. Similarly, IL-10 was stimulated by μsPEF to work as an anti-inflammatory cytokine. As a result, the necrotic tumor lesions were completely eradicated. But there is lack of proof to say the immue clearance process was tumor-specific.

To evaluate the safety of μsPEFs *in vivo*, porcine vital signs were monitored before treatment, during treatment, 1 hour post treatment and 24 hours after treatment. μsPEFs showed no damage on hemodynamic, pulmonary and renal function, but an impact on liver function. The aspartate aminotransferase, alanine amiontransferase and alkaline phosphatase increased 24 hours after μsPEFs which was the consequence of necrosis of the liver tissue covered by electrode.

To optimize the treatment parameters, the further measurement of the dielectric constant in the liver and tumor should be completed. μsPEF protocol should be individually decided on the basis of the better understanding of dielectric constant changes of liver tissue with different disease such as tumor, cirrhosis, hepatitis, parasite, ascites or congestion et al.

## Summary

As a locoregional minimum invasive treatment, μsPEFs can ablate HCC and avoid the abdominal laparotomy by a percutaneous approach guided by ultrasound without impact of hemodynamic, pulmonary and renal function. Providing predictable necrosis and low complication, μsPEFs can be used repeatedly as a palliative treatment for HCC unresectable or ablative therapies for post resection recurrence or pretransplant downstage therapy.

## Author Contributions

S.Z. and C.Y. designed the experiments; X.C., Z.R., C.L., F.G., D.Z. and J.J. performed the experiments; X.C., Z.R. and C.L. analyzed the data; X.C. and J.S. provided technical and material support. X.C. and Z.R. wrote the manuscript; all authors reviewed the manuscript.

## Figures and Tables

**Table 1:** Hemodynamic monitoring and pulmonary function before and after μsPEFs treatment

Parameter	Baseline	0h	6h	24h	p value
HR	126.0 ± 23	151.4 ± 7.8	134.0 ± 25.3	120.3 ± 30.1	0.8877
MAP (mmHg)	122.4 ± 4.9	140.81 ± 0.82	132.58 ± 1.62	126.77 ± 2.82	0.4827
Temp (°C)	36.02 ± 0.42	36.1 ± 0.44	35.9 ± 0.46	36.09 ± 0.32	0.9009
PaCO_2 _(mmHg)	29.3 ± 1.9	30.2 ± 2.9	30.4 ± 1.3	30.1 ± 1.2	0.7398
PaO_2 _(mmHg)	110.23 ± 12	119.4 ± 6.3	100.4 ± 12	121.4 ±10	0.5141
Oxygen saturation	100%	95%	98%	99%	1.000
CVP (cm H_2_O)	7.8 ± 1.3	8.1 ± 1.9	8.2 ± 1.4	7.6 ± 1.1	0.9122

Hemodynamic and pulmonary functions were monitored by ECG monitoring. HR, heart rate (times per minute); MAP, mean arterial pressure; Temp, core temperature; PaCO_2_, arterial carbon dioxide partial pressure; PaO_2_, arterial oxygen partial pressure; CVP, central venous pressure. A time course was presented by before treatment (baseline), during treatment (0 h), 6 hour post treatment (6 h) and 1 day after treatment (24 h). Data were expressed as mean ± SEM. The p value indicates statistical analysis between the baseline level and 24 h after μsPEFs treatment.

**Table 2:** Liver function and kidney function before and after μsPEFs treatment

Parameter	Baseline	0h	6h	24h	p value
ALT (U/L)	35.2 ± 3.4	36.2 ± 2.3	53.8 ± 2.8	125.6 ± 3.1	<0.001***
AST (U/L)	34.0 ± 4.9	62.0 ± 6.1	71.6 ± 7.9	139.6 ± 54.0	0.0034**
ALK PHOS (U/L)	208.8 ± 21.2	284.4 ± 21.8	268.0 ± 29.8	379.8 ± 38.1	0.0025**
Urine volume (ml/hr)	151.0 ± 21	140.6 ± 7.7	144.0 ± 20.3	150.3 ± 10.1	0.9610
BUN (mg/L)	8.52 ± 0.65	8.81 ± 0.82	8.58 ± 0.62	8.77 ± 1.02	0.7384
Creatinine (mg/L)	1.00 ± 0.05	1.00 ± 0.13	1.01 ± 0.11	1.05 ± 0.25	0.7512

ALT, alanine amiontransferase; AST, aspartate aminotransferase; ALK PHOS, alkaline phosphatase; Urine volume was urine output per hour; BUN was blood urea nitrogen. A time course was presented by before treatment (baseline), during treatment (0 h), 6 hour post treatment (6 h) and 1 day after treatment (24 h). Data were expressed as mean ± SD. The p value indicates statistical analysis between the baseline level and 24 h after μsPEFs treatment.
