# Author Correction: Preclinical Study of Locoregional Therapy of Hepatocellular Carcinoma by Bioelectric Ablation with Microsecond Pulsed Electric Fields (μsPEFs)

**DOI:** 10.1038/s41598-024-68637-8

**Published:** 2024-08-07

**Authors:** Xinhua Chen, Zhigang Ren, Chengxiang Li, Fei Guo, Dianbo Zhou, Jianwen Jiang, Xinmei Chen, Jihong Sun, Chenguo Yao, Shusen Zheng

**Affiliations:** 1 The Department of Hepatobiliary and Pancreatic Surgery, School of Medicine, The First Affiliated Hospital, Zhejiang University, Hangzhou, 310003 Zhejiang China; 2 Collaborative Innovation Center for Diagnosis and Treatment of Infectious Diseases, Hangzhou, 310003 Zhejiang China; 3 The State Key Laboratory of Power Transmission Equipment & System Security and New Technology, Chongqing University, Chongqing, 400030 China; 4 The Department of Pharmacy, Shandong University of Traditional Chinese Medicine, Jinan, 250014 Shandong China; 5 The Department of Radiology, Sir Run Run Shaw Hospital, School of Medicine, Zhejiang University, Hangzhou, 310003 Zhejiang China

Correction to: *Scientific Reports* 10.1038/srep09851, published online 30 April 2015

This Article contains errors in Figure 2.

As a result of errors during figure assembly, some of the panels in Figure 2d are incorrect. The Authors reviewed the original data and confirmed that the following panels were affected: the second image for µsPEFs 20 days group, the first and the second image for µsPEFs 28 days group, the second image for sham 28 days group, and the second image for sham 32 days group. The figure did not include images for the control and sham groups at 60 days, since, due to reaching the humane endpoint, these animals were sacrificed at 50-day mark.

The corrected Figure 2 and its accompanying legend appear below.

**Figure 2:**
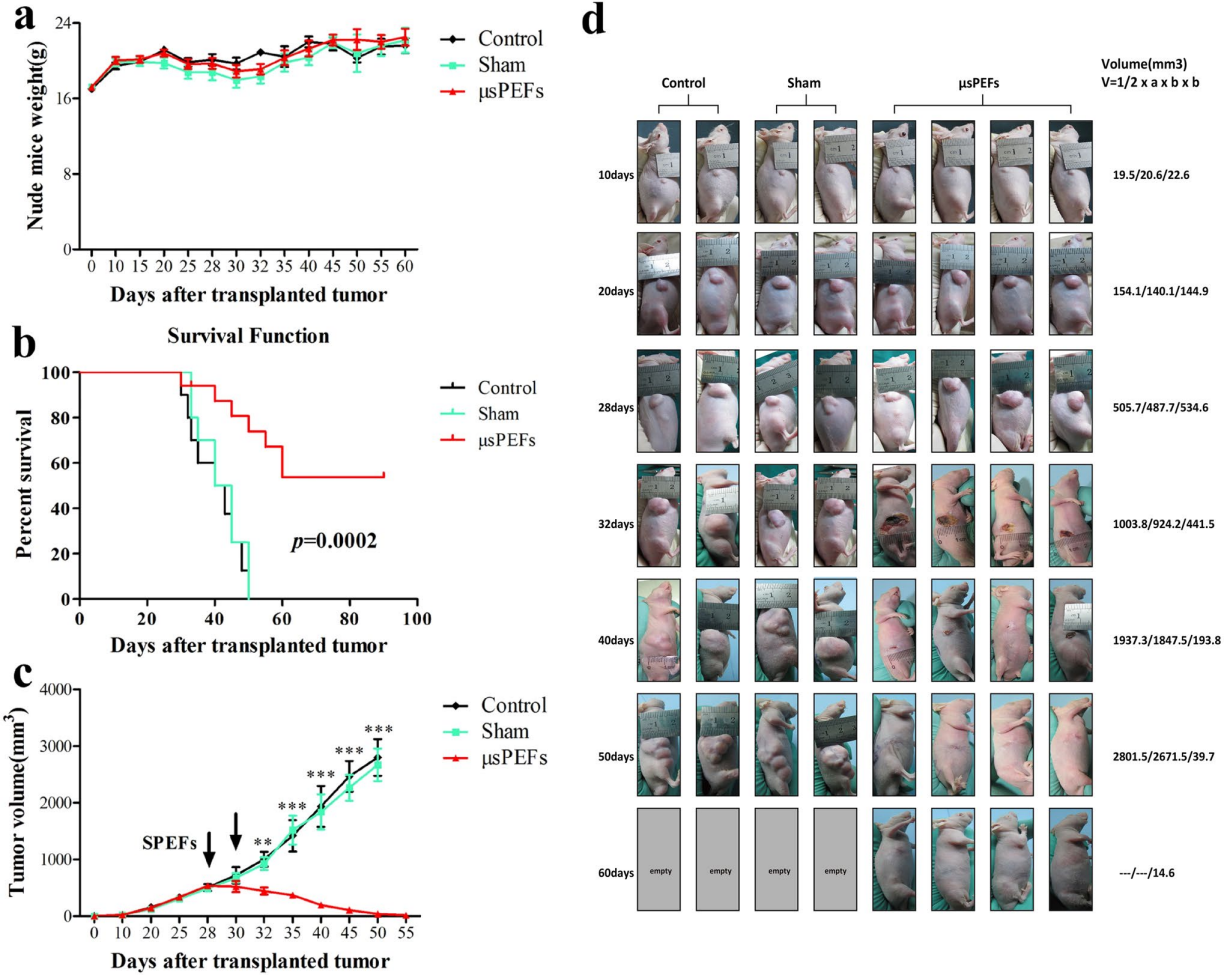
Robust efficacy of μsPEFs against Hep3B xenografts in nude mice. (**a**) The weights of nude mice from the different groups at different time points after transplanted tumor. The weight was present as mean ± SEM, n = 8. (**b**) Kaplan–Meier survival curve of nude mice implanted with Hep 3B tumor in the different groups (n = 8). (**c**) The changes of tumor volume in nude mice from the different groups at different time points after transplanted tumor. The tumor volume was presented as mean ± SEM, n = 8. **p < 0.01 or ***p < 0.001 indicated significant differences in μsPEFs treated group versus the control and sham groups. The arrows indicated twice μsPEFs on day 28 and day 30. (**d**) The dynamic process of μsPEFs ablation tumor compared with the control and sham groups. The volume presented as mean tumor volume (mm^3^) at each time point.

